# Ethical Considerations for safeguarding human participants in pandemic research: a rapid review protocol

**DOI:** 10.12688/hrbopenres.13053.2

**Published:** 2020-07-20

**Authors:** Lydia O'Sullivan, Ronan P. Killeen, Peter Doran, Rachel K. Crowley

**Affiliations:** 1 UCD School of Medicine, University College Dublin, Dublin 4, Ireland; 2 HRB Trials Methodology Research Network, Áras Moyola, National University of Ireland, Galway, Ireland; 3 UCD School of Nursing, Midwifery and Health Systems, University College Dublin, Dublin, Dublin 4, Ireland; 4 Research Ethics Committee, St Vincent's University Hospital, Dublin 4, Ireland

**Keywords:** COVID-19, pandemic, research ethics, clinical trials, case studies

## Abstract

COVID-19 is a respiratory disease caused by a coronavirus, designated SARS-CoV-2, which is responsible for a global pandemic in 2020. Public interest in this disease has led to the publication of thousands of articles in the medical literature in a very short timeframe. It is imperative that medical research into COVID-19 is conducted quickly and safely, and that due reference is given to the ethical considerations enshrined in the ICH GCP guidelines, according to the Declaration of Helsinki.

In order to review the reporting of ethical considerations in these papers, we hereby propose a protocol for a systematic review of COVID-19 papers up to April 14 ^th^ 2020. The search criteria proposed for the review are based upon what would be a reasonable search conducted by a lay member of the public with access to PubMed.gov. Institutional Research Ethics Committees (RECs) face significant challenges in providing thorough and timely ethical review during the COVID-19 pandemic. It is proposed to publish the findings of this rapid review along with a summary of an institutional REC response to the challenges of reviewing and approving clinical research proposals in the time of a pandemic.

## Introduction

In December 2019 the first reports of a novel coronavirus, now designated SARS-CoV-2, emerged from China ^1^. Since then SARS-CoV-2 has spread across the globe and is the agent responsible for a viral pneumonitis called COVID-19 ^2^. Its virulence, the rapidity of its spread and the lack of either a vaccine or a treatment have led to the adoption of unprecedented public health measures worldwide ^3^; and to high levels of interest and anxiety in the general population ^4^.

The international medical and scientific research community has responded to the COVID-19 pandemic by generating a huge amount of literature to which the general public has access ^5^. The COVID-19 related publications range from commentary and case reports to epidemiological studies and clinical trials. It is the policy of many journals during the pandemic to offer rapid review in order to make reports and research on COVID-19 available quickly ^6^. However, London and Kimmelman have expressed concern that scientific and methodological research standards will fall in the urgency to produce results ^7^. Similarly, the authors have observed considerable variability in the reported ethical review of the studies published and seek to conduct a systematic review of this question – how well are ethical disclosures ethical disclosures reported in the COVID-19 literature? Spector-Bagdady et al pointed out that clinicians and medical centres are faced with difficult decisions regarding which trials to participate in and are faced with challenges with implementing both an ethical and efficient recruitment method for COVID-19 trials ^8^. Tansey and colleagues also highlighted the importance of having a framework in place for ethical review in an emergency situations ^9^. Along with the results of this Rapid Review, a description will be provided of the response of an Irish Ethics Committee to the challenges presented by the COVID-19 pandemic. The Standard Operating Procedures of this Ethics Committee will be presented, along with how these were adapted to ensure thorough and timely ethical review during the COVID-19 pandemic.

## Protocol

### Protocol design and registration

The protocol is designed in line with the PRISMA-P checklist for systematic review ^10, 11^. The protocol is not eligible for registration on the PROSPERO database, as the question of interest relates to reporting and methodology rather than to a clinical outcome for participants.

The ethical standards of interest described by Yank and Rennie ^12^ will be used. These include an assessment of the standards agreed by the International Committee of Medical Journal Editors ^13^ following the Declaration of Helsinki ^14^; the report of consent obtained for the study and the report of ethical review of the study.

The search parameters are: Pubmed search of the term “COVID-19” with the filters of Free full text and English language publication – the rationale for this search is that it would be reasonable for a lay member of the public to conduct such a search. No start date is applied and the finish date of the search is April 14 ^th^ 2020.

### Search record and outcome management

The bibliography of the search will be saved and the assessors will use an Excel spreadsheet to record excluded and included studies, using the reference number from the search bibliography. Papers may be included if they are defined by an assessor as a case report, case series, observational study or clinical trial. Papers will be excluded if they are classified as a commentary, editorial, review, guideline, non-clinical report, cell-based study, animal study, epidemiologic study or report of a mathematical model. Two reviewers will conduct the initial assessment (half of the papers each, identified by the initial search). The reviewers will confer where there is any uncertainty.

Two independent reviewers will conduct a bias assessment. Each independent reviewer will assess 10 studies each for each initial assessor, identified randomly from the search bibliography (20 papers in total by each independent assessor). A threshold of 20% discrepancy between initial and independent assessors has been set to determine whether a further methodological revision is required. A further five records each will be reviewed following this initial method assessment.

For case studies and case series, the assessor will record: the first three authors; journal; study title; whether written consent reported; if not written consent whether oral consent reported or whether not available (i.e. patient deceased); whether written consent to publish provided to journal; the wording of consent; the nature of identifiers reported (such as location, occupation etc); whether the article included a statement of compliance with International Conference on Harmonisation (ICH) good clinical practice (GCP)/Declaration of Helsinki standards.

For observational studies and clinical trials, the assessor will record: the first three authors; journal; study title; whether written consent reported; the wording of consent; if not written consent whether oral consent reported; review by research ethics committee; whether that committee is identifiable on line; whether the article included a statement of compliance with ICH GCP/Declaration of Helsinki standards and in the case of a clinical trial, whether the trial was registered.

### Analysis

The outcome is the proportion of papers where informed consent to publish (in case studies and series) and for inclusion in a clinical research study as well as publication (observational studies and clinical trials) was recorded. An assessment of the role of ethics committees will be included; specifically, whether ethics committee approval was sought and whether requirement for consent was waived by said committee and whether there is a geographical or publication pattern associated with the reported role of the ethics committee.

This is a methodologic review; therefore, bias in interpretation of results is not relevant to this study. The proposed assessment of the ethics reporting standard categorized by journal may identify a publication bias.

A quantitative summary of numbers of studies identified, and their classification by assessor, will be provided. A further summary of consent reported and the nature of that consent will be prepared. The proportion of studies reporting ethical review and including a statement of compliance with ethical guidelines for conduct of clinical research studies (observational study or clinical trial), will be included. A qualitative description of the measures to preserve anonymity of the participants will be made.

### Ethical review and lay opinion

This study will not be clinical in nature and does not meet the requirement for a research ethics committee review. In view of the nature of the study and the impact of ethical conduct of research on public confidence in research, it is proposed to share the final draft of the study manuscript with two lay volunteers for a lay perspective on the findings prior to submission for publication. The protocol will be made available for the public on the UCD COVID-19 website.

## Future dissemination and availability of data

The search bibliography and finalized Excel spreadsheets will be included as supplementary documents with the final publication. As a publication relating to COVID-19, it is anticipated that this will be open access. Should this not be the case the authors will make these files available on request to the corresponding author.

## Conclusions

The purpose of conducting this review is to describe the existing publication status for ethical declarations in COVID-19 clinical research and discuss the potential impact on public trust in clinical research. It is proposed to include the findings with a description of how n Irish Research Ethics Committee conducted ethical assessment both expeditiously and safely in a time of pandemic; in order to maintain ethical standards, maximize public access to participation in clinical trials and research, and maintain public confidence in future research studies and their findings.

## Data availability

### Underlying data

No data are associated with this article.

### Reporting guidelines

Figshare: PRISMA-P checklist for ‘Protocol for systematic review of ethical declarations made in clinical publications concerning COVID-19’. https://doi.org/10.6084/m9.figshare.12249224.v1 ^11^.

The completed PRISMA-P checklist is available under the terms of the Creative Commons Attribution 4.0 International license (CC-BY 4.0).
