# Supplementary material for: Ethical Considerations for safeguarding human participants in pandemic research: a rapid review protocol
Source: HRB Open Res. 2020 Jul 20;3:22. Originally published 2020 May 13. [Version 2] doi: 10.12688/hrbopenres.13053.2 (PMC7424914; doi:10.12688/hrbopenres.13053.2)
Supplement: Peer review history

# Peer review: Reviewer response for version 2

**DOI:** 10.21956/hrbopenres.14214.r27696

**Published:** 2020-08-12

**Authors:** Bethany Spielman

**Affiliations:** 1 Southern Illinois University, Carbondale, IL, USA

I am satisfied with the changes; it can be indexed

Is the study design appropriate for the research question?

Yes

Is the rationale for, and objectives of, the study clearly described?

Yes

Are sufficient details of the methods provided to allow replication by others?

Yes

Are the datasets clearly presented in a useable and accessible format?

Not applicable

Reviewer Expertise:

Bioethics and law of human subjects research.

I confirm that I have read this submission and believe that I have an appropriate level of expertise to confirm that it is of an acceptable scientific standard.

# Peer review: Reviewer response for version 2

**DOI:** 10.21956/hrbopenres.14214.r27697

**Published:** 2020-08-04

**Authors:** Linda Biesty

**Affiliations:** 1 School of Nursing and Midwifery, National University of Ireland Galway, Galway, Ireland

Thank you to the team for updating the protocol and providing Version 2. I wish you all the very best with this review.

Is the study design appropriate for the research question?

Yes

Is the rationale for, and objectives of, the study clearly described?

Partly

Are sufficient details of the methods provided to allow replication by others?

Partly

Are the datasets clearly presented in a useable and accessible format?

Not applicable

Reviewer Expertise:

Qualitative research, trial methodology, evidence synthesis (qualitative and quantitative).

I confirm that I have read this submission and believe that I have an appropriate level of expertise to confirm that it is of an acceptable scientific standard.

# Peer review: Reviewer response for version 1

**DOI:** 10.21956/hrbopenres.14149.r27481

**Published:** 2020-06-10

**Authors:** Bethany Spielman

**Affiliations:** 1 Southern Illinois University, Carbondale, IL, USA

This can be a useful study. I look forward to reading it upon completion. The study could be improved by addressing several issues described below:

**Introduction**

 The following literature should be engaged/cited:

 London and Kimmelman (2020) ^1^; Spector-Bagdady *et al. *(2020) ^2^; Tansey *et al*. (2010) ^3^.

**Abstract**

 The abstract states that the study will publish a summary of the ethics committee responses to challenges of reviewing and approving protocols during a pandemic, but the protocol does not have a way of identifying what those challenges are or what committee responses to the pandemic situation have been. Some committees may always have been lax; the pandemic situation may not be more lax than their baseline. 

**Protocol**

 Protocol Design and Registration: 

 Please consider adopting completely Yank's categories for describing informed consent. Or, alternatively, explain: a) why you are not doing so and b) how readers should compare Yank's outcomes to yours. Comparison could be an important step in understanding pandemic constraints/responses if you make a greater effort to make your study comparable to Yank's.

 "Search Record and Outcome Management"

 For case studies and case series, the "wording of consent" will be recorded. It will not be recorded for observational studies and clinical trials. Why not?

**Conclusions**

 One of the stated purposes of the study is to describe the potential impact on public trust in clinical research. What are you going to ask lay people at the end of the study that will help determine that impact? Do you have enough lay people to say much? Is this really a second phase of the study?

 The conclusion states that a "description of how ethical assessment can be expedited safely in a time of pandemic...." will be included. The proposed study will not support any conclusions about whether the way things were done in the COVID 19 studies was expeditious or safe, or whether public access to participation was maximized. It will support conclusions about whether certain ethical standards were maintained (particularly if Yank's outcomes are used as a touchpoint); and it may (if the lay role is made more rigorous) support conclusions about public confidence.

Is the study design appropriate for the research question?

Yes

Is the rationale for, and objectives of, the study clearly described?

Yes

Are sufficient details of the methods provided to allow replication by others?

Yes

Are the datasets clearly presented in a useable and accessible format?

Not applicable

Reviewer Expertise:

Bioethics and law of human subjects research.

I confirm that I have read this submission and believe that I have an appropriate level of expertise to confirm that it is of an acceptable scientific standard, however I have significant reservations, as outlined above.

## References

[1] : Against pandemic research exceptionalism. *Science*.2020;368(6490) : 10.1126/science.abc1731 476-477 10.1126/science.abc1731 32327600

[2] : COVID-19 Clinical Trial Oversight at a Major Academic Medical Center: Approach of the Michigan Medicine COVID-19 Clinical Trial Committees. *Clin Infect Dis*.2020; 10.1093/cid/ciaa560 10.1093/cid/ciaa560 32392334PMC7239254

[3] : A framework for research ethics review during public emergencies. *CMAJ*.2010;182(14) : 10.1503/cmaj.090976 1533-7 10.1503/cmaj.090976 20530166PMC2950185

# Response

**Published:** 2020-07-15

**Authors:** Rachel Crowley

**Affiliations:** SVUH, Ireland

Thank you Bethany for your time in reviewing our protocol. We appreciate all of your comments and suggested changes. We have addressed each of the points you have raised and amended the protocol accordingly. 

Thank you for highlighting these publications. We have added them to the Introduction section. 

The final publication will include a description of the Standard Operating Procedures of a REC which will demonstrate the rigour of their review pre-COVID 19. The publication will also identify how they adapted their operational procedures to ensure timely and thorough ethical review during the COVID-19 pandemic. The Introduction and Conclusion have been amended to explain this.

The outcomes described by Yank and Rennie will be used. The wording in the ‘Protocol design and registration’ section has been amended to clarify this. 

The ‘wording of consent’ will be recorded for observational and clinical trials, so this sentence was amended accordingly. 

The purpose of the review is to determine to what extent COVID-19 publications report ethical declarations, given that the lack of these declarations may damage public trust in research. We have amended the wording of this section slightly to clarify this. The purpose of requesting two lay persons to review the manuscript was to incorporate their opinions, as well as the viewpoints of clinical researchers on the findings of this review. A larger exploration of lay opinions on COVID-19 research would indeed be valuable and interesting but it is unfortunately not within the scope of this research.

This wording referred to the description of the Standard Operating Procedures of an Irish Research Ethics Committee in pre-COVID 19 (please see amended text in the Introduction), which will be included in the publication. Along with this description, an account will be given of how this Ethics Committee adapted their practices to ensure timely and thorough ethical review despite the challenges presented by the COVID-19 pandemic. We have amended the wording of the Conclusion to clarify this.

Many thanks again Bethany.

# Peer review: Reviewer response for version 1

**DOI:** 10.21956/hrbopenres.14149.r27403

**Published:** 2020-05-26

**Authors:** Linda Biesty

**Affiliations:** 1 School of Nursing and Midwifery, National University of Ireland Galway, Galway, Ireland

I want to start by congratulating the team on their proposal. This is an important study and I was delighted to have the opportunity to offer some thoughts and comments. 

 Some points of feedback and comments:

**The Title of the Study:** I suggest that the title needs to be reviewed. At present it does not indicate clearly to a reader that “ethical declarations” relate only to the reporting of informed consent and ethics committee approval. The teams definition of ‘Clinical publications’ also needs to be explicit in the title and the ‘type’ of studies to be included needs to remain consistent throughout the protocol. 

**Abstract:** The last point in the abstract is not clear. What is meant by publishing the findings of this review “with” a summary of the IREC response to the challenges of reviewing and approving clinical research proposals in the time of pandemic? If the review authors intend to use the IREC response as a step in their interrogation of the findings this needs to be clear. 

**Introduction:** The review authors draw attention to the Torres-Salinas paper presenting “a global vision of the daily growth of scientific production on Covid-19 in various databases”. I was able to access only an English translation of the abstract of this paper, therefore, how it links to the amount of literature the general public can access, remains unclear. 

 The question identified at the end of this section (nature of ethical disclosure) is broader than the focus of the review as documented in other places (reporting of informed consent and ethics committee approval) this needs to be realigned. 

**Protocol: **Some rational needs to be provided as to why the review authors state that it is “reasonable for a lay member of the public to conduct such a search”. Is there evidence to suggest that the use of Pubmed (and Pubmed only) is appropriate? Have the different variations of the terms of COVID-19 been considered and how will the search account for this? Will this review include pre-prints and what considerations have been given to the time frame between the availability of a publication and its indexing in Pubmed? 

**Search record and outcome management: **Where does qualitative research fit within the inclusion / exclusion criteria?

**Analysis: **It is not clear what is meant by “a qualitative assessment”, this needs to be clarified. The analysis section notes that a qualitative description of the measures to preserve anonymity – however no reference is made to extracting data relating specifically to anonymity. 

**Ethical review and lay opinion: **Including a lay perspective in the review is welcome, however, the role of the 2 lay volunteers is not clear. Will the volunteers be asked offer their opinions in relation to the accuracy of the review, the rigour of your methods or the readability of the paper? 

**Conclusions: **The claims noted in this section need to be revisited. The purpose of this review noted on line 2 of this section denotes that the review will focus on “ethical standards in COVID-19 clinical trials” – this is not in keeping with the rest of the protocol. Nor does the protocol indicate how this review can highlight issues in relation to public trust. It is not clear how the review will provide a description of how ethical assessment can be expedited safely in a time of pandemic. Nor is it clear how the findings of this review will support the assumption that reporting ethical approval equates to maintaining ethical standards. The link between reporting the findings of this review and the “ … public’s confidence in future research and their findings” also needs to be reconsidered.

Is the study design appropriate for the research question?

Yes

Is the rationale for, and objectives of, the study clearly described?

Partly

Are sufficient details of the methods provided to allow replication by others?

Partly

Are the datasets clearly presented in a useable and accessible format?

Not applicable

Reviewer Expertise:

Qualitative research, trial methodology, evidence synthesis (qualitative and quantitative).

I confirm that I have read this submission and believe that I have an appropriate level of expertise to confirm that it is of an acceptable scientific standard, however I have significant reservations, as outlined above.

# Response

**Published:** 2020-07-15

**Authors:** Rachel Crowley

**Affiliations:** SVUH, Ireland

Thank you Linda for your time in reviewing our protocol. We appreciate all of your comments and suggested changes. We have addressed each of the points you have raised and amended the protocol accordingly.

Title was amended to reflect the reviewer’s comment and also because the authors feel that the review more accurately fits the Rapid Review methodology. Title now reads: *Ethical Considerations for safeguarding human participants in pandemic research: a rapid review protocol*

The wording of ‘clinical publications’ has been amended throughout to clarify and ensure consistency. 

A description of the response of an Irish Ethics Committee to the challenges presented by the COVID-19 pandemic will be provided in the Discussion section of the Rapid Review to complement the Rapid Review findings. Operational and logistical challenges in providing thorough and timely ethical review during the COVID-19 pandemic will be discussed. The Introduction and Conclusion were amended to clarify this.

The Torres-Sallinas reference was removed and a reference to an announcement on the PubMed website that over 50 publishers have made COVID-19 publications accessible was added.

The sentence at the end of the Introduction (nature of ethical disclosure) was amended to clarify scope of this review.

PubMed facilitated over 50 publishers in providing access to full-text publications relating to COVID-19: https://www.ncbi.nlm.nih.gov/pmc/about/covid-19/ so the rationale was that this platform would make these publications accessible to members of the public. A reference to this webpage has been added to the protocol (see above point). 

The purpose of this rapid review was to assess how well a representative sample of human research on COVID-19 adhered to ethical guidelines such as informed consent, REC review etc, rather than to perform an exhaustive search of all COVID-19 publications. For this reason, a single but comprehensive database was selected, which provides full-text access to lay people. Due to the fact that pre-prints have not undergone peer review, they will not be included in this review, as the intent is to assess peer reviewed articles. However, ‘Epub ahead of print’ articles will be included, as they have undergone peer review.

A search for ‘COVID-19’ in PubMed yields the following terms: "COVID-19"[All Fields] OR "severe acute respiratory syndrome coronavirus 2"[All Fields] OR "severe acute respiratory syndrome coronavirus 2"[All Fields] OR "2019-nCoV"[All Fields] OR "SARS-CoV-2"[All Fields] OR (("Wuhan"[All Fields] AND ("coronavirus"[MeSH Terms] OR "coronavirus"[All Fields])) AND 2020[All Fields]). It was therefore felt that that the term ‘COVID-19’ would provide us with a broadly representative sample of COVID-19 research publications.

No filters will be applied other than ‘Free Full Text’ and ‘English Language’ so qualitative studies will be included. A initial search indicated that the qualitative COVID-19 studies were   observational in nature, so they will be included in this category. 

A review of whether publications record if REC approval was sought and whether the requirement for consent was waived by the REC will be conducted. If the information is available, a review will be conducted of the geographical or publication patterns also. We have removed the word ‘qualitative’.

Under ‘Search Record and Outcome Management’, one of the items that will be recorded is ‘nature of identifiers’. We have added ‘(such as location, occupation etc)’ to clarify the meaning of this.

The purpose of requesting two lay persons to review the manuscript was to incorporate their opinions, as well as the viewpoints of clinical researchers on the findings of this review. We have amended this sentence to clarify.

The final publication will include a description of the Standard Operating Procedures of a REC which will demonstrate the rigour of their review pre-COVID 19. The publication will also identify how they adapted their operational procedures to ensure timely and thorough ethical review during the COVID-19 pandemic. The Conclusion has been re-worded to address this comment. 

Many thanks again Linda.
